# Patterns and predictors of periodontal disease and tooth loss among users of smokeless tobacco

**DOI:** 10.1186/s12903-023-03087-8

**Published:** 2023-06-27

**Authors:** Syed Muazzam Nasir, Tajwer Sultana, Shahkamal Hashmi, Mansoor Ahmed

**Affiliations:** 1 Department of Biosciences, Shaheed Zulfiqar Ali Bhutto Institute of Science and Technology, Karachi, Pakistan; 2 School of Public Health, Dow University of Health Sciences, Ojha Campus, Gulzar-e-Hijri Road, Scheme 33, Suparco Road, Karachi, Pakistan

**Keywords:** Periodontal disease, Community periodontal index, Gingival recession, Tooth loss, Smokeless tobacco

## Abstract

**Background:**

Smokeless tobacco (SLT) products of several different types are widely used in several South Asian countries including Pakistan. These products are consumed in different forms and with different names. The study aims to determine adverse effects of the SLT consumption on periodontal tissues.

**Methods:**

This cross sectional study was conducted in Karachi, Pakistan. It recruited 377 users of (SLT) with 231 males (61.3%) and 146 females (38.7%) of age 15 to 45 years. After obtaining informed consent, quantitative data were collected via a questionnaire followed by intra oral clinical examination to determine presence of periodontal diseases using community periodontal index (CPI). To determine the association between periodontal diseases and smokeless tobacco consumption habits, Chi Square test was conducted.

**Results:**

Gingival recession (Class II-IV) (65.8%) was the most prevalent periodontal disease among SLT users. CPI score was high (CPI score 3 and 4) in 31.3% participants, whereas tooth loss was found in 21%. Among types of SLT products, gutka (28.6%) and betel quid (23.3%) were most commonly used. Using SLT for five or more years was found to be associated with a high CPI score, gingival recession (Class II-IV), moderate to severe tooth mobility, and presence of tooth loss.

**Conclusions:**

The study found statistically significant association between duration in years of using SLT and periodontal disease including gingival recession, tooth mobility and tooth loss. However, no significant results were found between retention during single use and frequency of SLT use per day. However, the link of these factors with the periodontal disease cannot be eliminated.

## Background

Periodontal disease is an umbrella term for gingivitis and periodontitis and describes a chronic inflammatory condition of tooth-supporting tissues [1]. It is also characterized by gingival recession, bleeding on probing or with physiological pressure (that is exerted during mastication), drifting of tooth, and increased tooth mobility (if left untreated might lead to tooth exfoliation) [2]. Most forms of periodontal disease are chronic inflammatory conditions that progress intermittently [3]. Bacterial plaque accumulation is the primary cause of periodontal disease [1]. However, several other local and systemic predisposing factors can modify the occurrence and progression of the disease [4].

Smoking and consumption of smokeless tobacco (SLT) are among the most common predisposing risk factors of periodontal health deterioration and oral cancer [1, 5]. Literature highlights effects of using SLT on oral and periodontal tissues [6]. In the absence of plaque, gingival recession may be caused by mechanical or chemical injury to periodontal tissues [7]. Since SLT products are high in concentration of nitrosoproline, nitrosodiethanolamine and other tobacco-associated nitrosamines, these can be a major source of chemical injury to mucosa and gingival tissues that are chronically exposed to it [8]. Hence, SLT containing chemicals may cause recession and it can be classified as a form of periodontal disease. However, recession due to the mechanical irritation from the SLT may not be classified as periodontal disease.

SLT is used with great diversity in different parts of the world that complicates the understanding of its use and impact. SLT products are widely available in two major forms: chewable tobacco and snuff. The habit of SLT consumption refers to use of unburned tobacco, in different forms that includes chewing paan (betel quid with tobacco), gutka (areca nut with tobacco), dipping and snuffing of naswar, loose leaf etc. The powdered form of tobacco (snuff) is also used which is finely cut or with addition of sweeteners and various flavoring agents [9]. According to the Pakistan Demographic and Health Survey 2017-18, 14.6% of men and 3.4% of women of age 15–49 years are consumers of any form of SLT. The most common types of SLT used in Pakistan are betel quid with tobacco (paan), gutka, naswar, dry packet, mawa/mainpuri and betel nut [10].

SLT consumption effects have been linked with oral cancerous and pre-cancerous conditions, however emerging data exhibit that this habit has harmful effects on gingival and oral tissues [11]. The initial adverse effects of SLT use are usually limited to oral cavity that may be mild to severe, such as alteration of taste, discoloration and extrinsic stains on teeth, tongue and oral mucosa, attrition, caries, gingival bleeding, and periodontal pocket formation [12]. Tobacco chewing habit also results in gingival recession, halitosis, gingival irritation, oral mucosal ulcers and sores [13]. Oral ulceration caused by SLT results in difficulty to consume spicy foods [14]. Weakening senses of smell and taste are also noticed [15]. SLT consumption for longer period results in moderate to severe adverse effects such as severe periodontitis and gingival recession that ultimately leads to tooth mobility and subsequently to tooth loss, severe bone loss, pre-cancerous and cancerous lesions [16].

Use of SLT is found to be associated with cancer of head and neck [17], pancreas [18], oral periodontal diseases and mucosal lesions [19, 20]. Several studies have documented association of periodontal diseases with the use of SLT [21, 22]. Earlier studies that are conducted in the United States and Sweden show that use of SLT causes increased incidence of gingival recession [6, 23]. Whereas more recent studies did not show any such association [7]. However, studies conducted in India, Bangladesh and Thailand exhibit that users of SLT are relatively at higher risk of periodontal diseases as compared to non-users [22, 24, 25]. Pakistan is among some of the countries where the use of SLT is culturally acceptable. Most SLT users in the country believe that it is not harmful or is less harmful than tobacco smoking. Therefore, it is essential to explore the prevalence of using various forms of SLT and its possible relationship with periodontal diseases in Pakistan. To the best of our knowledge, there is scarcity of evidence in this area from Pakistan. The objective of this study was to determine any association between SLT products with various periodontal diseases in Pakistan.

## Methods

### Study design and sample

In this cross sectional study, a total of 377 individuals were included, n = 238 males (63%) and n = 139 females (37%), of age 15–45 years with the habit of SLT consumption, reporting to outpatient department of Fatima Jinnah Dental College Hospital located at Azam Town Adjacent to Phase 1 Defence Housing Authority, Karachi, Pakistan. The continuous sampling method was used. The participants of the study initially filled questionnaire followed by clinical examination for the evaluation of periodontal disease and prevalence of tooth loss. To see the association between variables we used the following equation to calculate the sample size based on results of a previous study [19]:

$$\frac{n={Z^2} \cdot p \cdot (1-p)}{{d^2}}$$

Rounding up to the nearest whole number, the minimum sample size required is 302. To account for a 20% non-response rate, 377 individuals were sampled.

### Inclusion and exclusion criteria

Inclusion criteria were individuals aged between 15 and 45 years, smokeless tobacco users, and having at least one tooth present in both arches. Whereas the exclusion criteria were non smokeless tobacco users, patients with any uncontrolled systemic diseases, tobacco smokers, individuals with drug history of phenytoin, steroids etc., pregnant and lactating females, and edentulous patients.

### Data collection

Before the start of the study, it was approved by Institutional Ethical and Scientific review committee of Institutional Ethical Review Board of Shaheed Zulfiqar Ali Bhutto Institute of Science and Technology, Karachi, Pakistan (IERB(4)/SZABIST-KHI(MPH)/1,740,135/190,002). After obtaining written informed consent from each participant of the study, data were collected in March 2019 using a structured questionnaire. It was self-administered under the supervision of the first author (SMN). The items included were sociodemographic questions such as age, gender, marital status, level of education, and brushing habits. Questions on SLT included its types, duration (in years), frequency of use per day and duration of retention in mouth during single use of SLT (in minutes).

### Clinical examination

Following the questionnaire, the participants of the study underwent oral clinical examination under good lighting conditions on a dental chair. The oral clinical examination included evaluation of periodontal health by evaluating periodontal pockets, gingival recession, tooth mobility, furcation involvement and teeth loss due to mobility. The instruments used for oral examination were mouth mirror, tweezers, CPI probe, explorer and Naber’s probe. The periodontal disease status of participants was evaluated using CPI probe with Community Periodontal Index (CPI) that includes evaluation of Periodontal Probing Depth (PPD). It is a lightweight probe with a ball tip of 0.5 mm. Additionally, the clinical attachment loss (CAL) was also assessed for indexed teeth that are defined in the criteria by WHO [26]. In case where indexed teeth was absent in a particular sextant, all the teeth of that sextant were examined for CAL.

The participant’s teeth were divided into six different sextants and in each sextant following teeth were evaluated 17, 16, 11, 26, 27, 47, 46, 31, 36, and 37 based on criteria for evaluation [27]. The participants with CPI score 0 means they have healthy periodontium and the CPI score 1 means there was gingival bleeding on using CPI probe. The CPI score 2 means presence of calculus felt either through instrumentation or visual examination and the CPI score 3 showed periodontal pocket depth of 4–5 mm. The CPI score 4 showed periodontal pocket depth of more than 6 mm. The CPI scores of 0,1 and 2 were grouped as absence of periodontal pockets while CPI score 3 and 4 were grouped as presence of periodontal pocket. Clinical Attachment Loss (CAL) was also assessed using CPI Probe by calculation of the distance between cementoenamel junction to the base of sulcus or pockets.

Teeth Mobility measurement was carried out by holding the tooth firmly between the handles of two metallic instruments and effort was made to move it in all directions. Following criteria was used to assess teeth mobility: Grade 0 for no mobility, Grade 1 for slight mobility less than 1 mm, Grade 2 mobility of 1–2 mm and Grade 3 for mobility of more than 2 mm [20]. For this study, Grade 0 and 1 were categorized as no/mild tooth mobility and Grade 2 and 3 were categorized as moderate/severe tooth mobility. Naber’s probe was used for evaluation of furcation involvement. Scoring criteria modified by Loesche et al. was used [20]. Score 0 indicates no furcation involvement, score 1 indicates slight indentation, score 2 indicates pronounced indentation, score 3 indicates through-and-through penetration of Naber’s probe but filled with soft tissue and might not be visible, and score 4 indicates through-and through-penetration and furcation clinically visible. The participants with score 0 and 1 were categorized as having no/mild furcation involvement while 2–4 were grouped as having moderate/severe furcation involvement. Gingival recession is exposure of root surface of the tooth due to apical shift/migration of marginal gingiva. Gingival recession of the tooth was assessed using the CPI probe and categorized according to the criteria by P.D Miller [21]. For this, linear measurements were done from the cement enamel junction to free margin of the gingival crest, using periodontal probe.

### Statistical analysis

Statistical analysis of the data was performed using the Statistical Package for the Social Sciences (SPSS) version 21. Data were presented as frequencies and percentages. Pearson’s Chi-square test was used to analyze any relationship between use of smokeless tobacco and different periodontal diseases. Logistic regression was used to further analyze variables that showed statistical significance in the Chi-square test. A p-value of < 0.05 was considered statistically significant.

## Results

Table 1 shows sociodemographic characteristics and use of SLT among study participants. The mean age of participants was 32.64 ± 8.609 ranging from 15 to 45 years. Out of 377 participants, 64.2% were of age 30 years or above, 61.3% were males, 44.29% had secondary education, and 40.58% had habit of brushing once or more than once a day. Regarding types of SLT consumption, betel quid with tobacco (paan), gutka and naswar were most commonly used. For duration, 55.43% were using for five years or more, 49.1% were keeping any type of SLT for five minutes or more, and 60.5% were using it 5 times or more in a day.

**Table 1:** Sociodemographic characteristics and use of smokeless tobacco

Variables	Categories	Frequency (N 377)	Percentage
Age	Under 30 year	135	35.8
	30 years or above	242	64.2
Gender	Male	231	61.3
	Female	146	38.7
Education	Primary	72	19.09
	Secondary	167	44.29
	Tertiary	138	36.6
Brushing Habits	Once or more in a day	153	40.58
	Occasionally	141	37.4
	Never	83	22.01
Type of smokeless tobacco (SLT)	Betel quid with tobacco (paan)	88	23.3
	Gutka	108	28.6
	Nawar	67	17.8
	Dry packet	22	5.8
	Mawa/mainpuri	68	18
	Betel nut	13	3.4
	others	11	2.9
Using SLT for years	Less than 5	168	44.6
	5 or more	209	55.4
Retention of SLT in mouth in minutes	Less than 5	192	50.9
	5 or more	185	49.1
Frequency of using SLT in a day	Less than 5	149	39.5
	5 or more	228	60.5

Table 2 shows periodontal disease on clinical examination among study participants. Gingival recession of Class II-IV (65.8%) was found to be the most prevalent in SLT users among all periodontal diseases. Community periodontal index (CPI) score was high (CPI score 3 and CPI score 4) 31.3% and moderate/severe furcation involvement was found in only 4.2% participants. Moderate to severe tooth mobility was present in 14.1% whereas tooth loss was found in 21% of SLT users.

**Table 2:** Clinical examination of study participants

Variables	Categories	Frequency (N 377)	Percentage
CPI score	1 and 2	259	68.7
	3 and 4	118	31.3
Gingival recession	Class I	129	34.2
	Class II - IV	248	65.8
Furcation involvement	no/mild	361	95.8
	present	16	4.2
Tooth mobility	no/mild	324	85.9
	moderate/severe	53	14.1
Tooth loss	absent	298	79.0
	present	79	21.0

Table 3 shows predictors of CPI score, tooth mobility and tooth loss among users of SLT. High CPI scores were found among users of betel quid, gutka, and mawa/mainpuri compared to other products. Tooth mobility and tooth loss were also most frequently documented among users of betel quid. Using SLT for five or more years was found to be associated with a high CPI score (P value < 0.001), moderate to severe tooth mobility (P value < 0.001), and presence of tooth loss (P value < 0.001). No significant association was found for retention time of SLT in mouth and frequency of its use in a day.

**Table 3:** Predictors of CPI score, tooth mobility and tooth loss

SLT consumption		CPI score			Tooth mobility			Tooth loss		
		1 & 2	3 & 5	P value	No/mild	Moderate/severe	P value	Absent	Present	P value
Betel quid with tobacco (paan)		57	31		73	15		69	19	
gutka		84	24		99	9		94	14	
Nawar		48	19		59	8		54	13	
Dry packet		15	7		20	2		18	4	
Mawa/mainpuri		45	23		59	9		52	16	
Betel nut		3	10		4	9		3	10	
others		7	4		10	1		8	3	
Using SLT for years	Less than 5	146	22	< 0.001	162	6	< 0.001	154	14	< 0.001
	5 or more	113	96		162	47		144	65	
Retention of SLT in mouth in minutes	Less than 5	132	60	0.983	162	6	0.555	155	37	0.413
	5 or more	127	58		162	47		143	42	
Frequency of using SLT in a day	Less than 5	109	40	0.132	133	16	0.134	120	29	0.565
	5 or more	150	78		191	37		178	50	

Table 4 shows predictors of gingival recession and furcation involvement among users of SLT. Gingival recession (Class II-IV) was most frequently documented among users of betel quid, gutka, and mawa/mainpuri. Using SLT for five or more years was significantly associated with gingival recession (Class II-IV). No significant association was found for retention time of SLT in mouth and frequency of its use in a day.

**Table 4:** Predictors of gingival recession and furcation involvement

SLT consumption		Gingival recession			Furcation involvement		
		Class I	Class II -IV	P value	no/ mild	moderate/ severe	P value
Betel quid with tobacco (paan)		24	64		84	4	
Gutka		40	68		103	5	
Nawar		31	36		65	2	
Dry packet		5	17		21	1	
Mawa/mainpuri		24	44		65	3	
Betel nut		2	11		12	1	
others		3	8		11	0	
Using SLT for years	Less than 5	86	82	< 0.001	166	2	0.008
	5 or more	43	166		195	14	
Retention of SLT in mouth in minutes	Less than 5	69	123	0.473	185	7	0.557
	5 or more	60	125		176	9	
Frequency of using SLT in a day	Less than 5	54	95	0.503	143	6	0.866
	5 or more	75	153		218	10	

Chi-square analysis revealed that periodontal damage was significantly associated with duration of using SLT. These variables were further analyzed using logistic regression to see their association with use of SLT for five years or more. The results are presented in Table 5, which show that only gingival recession class II to class IV was significantly associated with use of SLT for five year or more.

**Table 5:** Logistic regression model showing relationship between use of SLT for five years or more and different periodontal diseases

Variables	OR	95% CI	P-value
CPI score			
1 and 2	1	Ref	
3 and 4	1.074	0.478–2.403	0.861
Gingival recession			
Class I	1	Ref	
Class II - IV	1.901	1.033–3.455	0.042
Furcation involvement			
no/mild	1	Ref	
present	0.871	0.371–2.048	0.772
Tooth mobility			
no/mild	1	Ref	
moderate/severe	0.654	0.327–1.897	0.084
Tooth loss			
absent	1	Ref	
present	0.751	0.284–2.026	0.093

## Discussion

This study reports prevalence of periodontal diseases among SLT users in Pakistan. Similar results are reported from studies conducted in Bangladesh, India and Thailand [22, 24, 25]. Among South Asian populations including Pakistan, use of SLT is common. A study published in 2015 reported that Pakistan and India together have estimated 100 million SLT users [28]. In India, 35–40% of tobacco consumption is found to be in SLT form while in Pakistan studies and national demographic data have reported that around 21% men and 12% women are users of betel quid with or without tobacco [10, 28]. Although legislation in Pakistan prohibits manufacturing and distribution of SLT, it is still widely accessible by the people. Epidemiological studies about effects of SLT and its association with different diseases except for oral cancer and precancerous lesions and conditions, has not gained much attention in the country yet. Furthermore, in developing countries, irrational use of products without prior knowledge and lack of standard safety checks, such as SLT, is a huge concern [29]. Pakistan is among 11 countries that account for high prevalence of SLT use and global burden of diseases associated with it. Therefore, just like any clinical research, association of various periodontal and oral soft tissue diseases needs to be studied more according to local trends, traditions and behaviors of SLT consumption [28, 30].

This study recruited 377 SLT users. Among them, 231 were males (61.3%) and 146 were females (38.7%). Among types of SLT used, gutka, betel quid, and naswar were most commonly reported. Similar findings are reported in earlier studies [31]. While mawa & mainpuri, dry packet sachets, and betel nut were also reported in our population. We also found that majority of the users were chronic consumers of SLT, which is in line with the available literature [32]. It is understandable because SLT use in Pakistan runs in families and in all generations. Moreover, they do not consider it important to share their SLT history with their healthcare providers. Non-disclosure of using potentially harmful products with the healthcare providers is a matter of concern especially in developing countries [33]. This study also found that nearly half of the SLT users retain the product for over five minutes. Studies have reported that longer the retention of SLT in mouth, the more harmful effects can be [12]. Additionally, most of the SLT users consumed five or more doses in a day, which is also in line with an earlier study [34]. A few studies suggest that certain inflammatory mediators may be amplifying periodontal diseases. Mediators such as interleukin-1 (IL-1) and IL-8 have been linked to gingival and periodontal inflammation [35]. The main alkaloid in areca nuts i.e. arecoline, and the nicotine, are found in the majority of SLT products alter and control the inflammatory process by changing the amounts of prostaglandin E_2_ (PGE_2_), IL-1β, IL-β, IL-6, and TNF-α [36].

Gingival recession (Class II-IV) was found to be the most prevalent among periodontal diseases. It is consistent with the report from Atlanta [37]. CPI score was also high (CPI score 3 and 4) in 118 participants, which suggests those participants had periodontitis. High CPI scores and gingival recession (Class II-IV) were more commonly found among betel quid and gutka users as compared to SLT users of other types. Similar findings were documented by a study from Thailand [25]. Gingival Recession may also represent as a sign of periodontal disease, because gingival recession may be found with the presence of normal sulci and levels of interdental crestal bone that are not diseased or may be found as part of the periodontal disease pathogenesis where alveolar bone is lost [38]. Logistic regression analysis also revealed association between gingival recession (Class II-IV) and use of SLT for five year or more.

Statistically significant association between duration in years of using SLT and periodontitis (CPI score 3 and 4) was found. That indicates that greater duration in years of SLT usage results in higher prevalence of periodontitis and gingival recession (Class II-IV). Whereas no statistically significant association was found between periodontitis and frequency of use per day and retention during single use. However, it cannot be concluded that longer retention of SLT in mouth or frequency of its usage per day would not have deleterious effects on periodontal tissues. More studies are needed to understand this better. Clinical attachment loss (CAL), which is an important indicator of periodontal disease, was found in 32% of users with score of more than 3 mm. This data of clinical attachment loss indicates that 32% participants of all SLT users have untreated periodontal disease. Whereas moderate/severe furcation involvement was least prevalent among periodontal diseases.

Moderate to severe tooth mobility (Grade II & Grade III) and at least one tooth loss excluding absence of third molar was found more prevalent among betel quid, gutka, naswar and mawa & mainpuri users than users of other SLT forms. These findings are in line with the published literature [2]. Further analysis of the data showed statistically significant association between duration in years of use of SLT with tooth mobility and tooth loss. However, no statistically significant results were found between tooth mobility and frequency of use per day and retention during single use.

Analysis of this study suggests that prevalence of periodontal diseases including periodontitis, gingival recession, tooth mobility and tooth loss are high among SLT users in Pakistan. The study results suggested statistically significant association between duration in years of use of SLT and several periodontal diseases. This indicates that time duration in years a person is consuming SLT increases risk of severe periodontal disease. This may result in compromising periodontal health, symptoms associated with periodontal disease (bleeding, sensitivity, and halitosis), and inability to chew and perform initial digestive procedure of pulverizing the food into digestible form and subsequently compromising health and wellbeing. The findings of the study can be helpful for policy makers, and users of SLT as a warning signal to motivate them towards its cessation. The study demonstrates that consumption of various products of SLT may not only cause oral cancerous and pre-cancerous lesions but also certain conditions that are not lethal, yet may cause health compromising adverse effects.

The study has few limitations. The study was conducted in one city of Pakistan so its findings should not be generalized to the whole population of the country. Moreover, the prevalence of gingival recession is reported based on the consumption of SLT, which is locally pouched in the mouth. The full mouth periodontal status would give clearer illustration of the prevalence of gingival recession. Later, full mouth periodontal examination was carried out in the outpatient department and patients were referred to the periodontology department for further evaluation. Furthermore, periodontal parameters between retention and non-retention sites were not compared. Additionally, it could not be substantiated whether tooth loos was due to tooth mobility and periodontal disease. Lastly, future studies may also incorporate cotinine test to determine the level of nicotine, and radiographs that are highly effective in evaluating periodontal diseases and bone loss.

## Conclusion

The findings of the study can be helpful for policy makers, and users of SLT as a warning signal towards its cessation. The study demonstrates that consumption of various products of SLT may not only cause oral cancerous and pre-cancerous lesions but also some conditions that are not lethal, yet may cause health compromising adverse effects. The study found statistically significant association between duration in years of using SLT and several conditions including periodontitis, gingival recession, tooth mobility and tooth loss. However, no significant association was found between SLT, retention during single use and frequency of SLT use per day. Nevertheless, the role of these factors in contribution of the disease cannot be eliminated. Findings of this study add to the global statistics of harmful effects of SLT products. It is essential to conduct more studies to produce further scientific evidence, which can be disseminated to promote knowledge and awareness about harmful effects of using SLT.

## Data Availability

The data can be requested by contacting the corresponding author (MA) of this study.

## Abbreviations

SLT: Smokeless tobacco
CPI: Community periodontal index
CAL: Clinical attachment loss
PPD: Periodontal probing depth
